# Patient-centred interprofessional education in cancer care: a scoping review protocol

**DOI:** 10.1136/bmjopen-2024-089909

**Published:** 2025-02-18

**Authors:** Adis Šerifović, Tobias Fragner, Honja Hama, Kathrin Kirchheiner, Igor Grabovac

**Affiliations:** 1 Open Innovation in Science Center, Ludwig Boltzmann Gesellschaft, Vienna, Austria; 2 Department of Social and Preventive Medicine, Medical University of Vienna, Vienna, Austria; 3 Department of Radiation Oncology, Medical University of Vienna, Vienna, Austria

**Keywords:** ONCOLOGY, Interprofessional Relations, MEDICAL EDUCATION & TRAINING, Patient-Centered Care, Person-Centered Care

## Abstract

**Introduction:**

Cancer remains a major global health challenge, affecting millions annually and ranking as the second leading cause of death worldwide. The complexity of cancer treatment requires an interdisciplinary approach, connecting professionals from various fields to deliver personalised and integrated care. However, structural issues and insufficient interdisciplinary training can impede effective collaboration, which is why effective interprofessional education (IPE) is needed. This protocol depicts the planned procedures for a scoping review that aims to explore the role of IPE in enhancing interdisciplinary collaboration within oncology by mapping and synthesising the implementation, impact and evaluation strategies of patient-centred IPE programmes.

**Methods and analysis:**

This scoping review will be conducted in line with the Joanna Briggs Institute guidelines for scoping reviews. The research team will develop a comprehensive search strategy and apply it to the following databases: CENTRAL, CINAHL, Embase, MEDLINE, PsycInfo, Scopus and Web of Science . Additionally, we will search for grey literature (eg, using OpenDOAR) and contact relevant organisations for pertinent reports. Each database will be searched without date restrictions on 11 September 2024. In the first stage, eligibility criteria will be assessed through a blinded title and abstract screening, followed by a full-text review. The research team will then extract and synthesise data related to the scoping review questions, focusing on implementation, impact and evaluation strategies employed in the included studies.

**Ethics and dissemination:**

As this protocol does not involve collecting primary data, ethical approval is not required. The results of this review will be published in a peer-reviewed journal and disseminated through institutional websites and conferences.

**STRENGTHS AND LIMITATIONS OF THIS STUDY**

- This review will be conducted following the Joanna Briggs Institute guidelines, ensuring methodological rigour and transparency.
- No restrictions on language or location will be applied, allowing for a comprehensive synthesis of global research on interprofessional education (IPE) in oncology.
- The search strategy includes both peer-reviewed and grey literature, minimising the risk of publication bias and ensuring a broad coverage of evidence.
- Studies will be selected and reviewed independently by multiple researchers, enhancing the reliability of the selection process.
- This scoping review will use a structured data extraction and analysis process to systematically map and summarise the implementation, impact and evaluation strategies of patient-centred IPE programmes.

## Introduction

 Cancer is the second leading cause of death worldwide, with an estimated nearly 10 million deaths in 2020 alone.^1^ In cancer care, interdisciplinary approaches are crucial for improving patient outcomes and enhancing the efficiency of healthcare delivery through interdisciplinary training via interprofessional education (IPE). These issues, such as rigid hierarchies, lack of standardised communication protocols and undefined interdisciplinary roles, can be mitigated by fostering better collaboration and communication and ultimately improving patient care outcomes.^2^ Interdisciplinary teams often include a wide range of professionals, such as physicians from various specialities, nurses and allied health professionals (eg, psychologists, dietitians, social workers, occupational therapists, speech therapists and physiotherapists). Given the complexities associated with cancer treatment, these teams aim to provide comprehensive and multifaceted treatment and support to patients and their caregivers. This includes physical, psychological and social care, as well as personalised treatment plans that address the unique needs of each patient and their families.

However, implementing interdisciplinary approaches is connected with significant challenges. Structural problems, such as rigid hierarchies, a lack of understanding of interdisciplinarity and the remit of other fields or professions, can hinder collaboration in healthcare settings, as evidenced by Aebersold *et al*,^3^ who found that the absence of standardised communication protocols among healthcare teams led to errors in chemotherapy administration. Rigid hierarchies created an environment where team members hesitated to speak up or question decisions, leading to critical information being overlooked. Additionally, the lack of understanding of interdisciplinary roles meant that professionals were not always aware of who to communicate with regarding specific issues, further complicating the process. These problems often stem from traditional medical training programmes that prioritise discipline-specific education, creating silos in healthcare teams and limiting professionals’ ability to collaborate effectively across disciplines. As highlighted by Aebersold *et al*^3^, such training can lead to fragmented communication, inadequate role clarity and errors in patient care.

IPE has been identified as a key strategy for improving collaboration among healthcare workers. It enhances collaborative learning and teamwork across various healthcare disciplines, improving patient outcomes in fields such as primary care, paediatrics, and surgery.^4^ ^5^ Non-oncological IPE programmes, such as those implemented in surgery and paediatrics, have demonstrated reduced medical errors, improved communication and better patient safety.^6^ These programmes typically vary in structure and duration, ranging from short-term workshops to long-term integrated training, depending on the specialty and the specific learning objectives. Incorporating these insights into oncology-related IPE programmes can help improve the interdisciplinary collaboration needed for complex cancer care.

IPE programmes foster mutual respect and understanding among various professional groups, enhancing teamwork and communication.^7^ Studies have demonstrated that IPE can improve healthcare professionals’ attitudes, knowledge and skills, ultimately leading to better patient care.^6^ For example, Papadakos *et al*^8^ showed that an IPE programme significantly increased the competence of oncology healthcare providers in handling difficult conversations with patients, highlighting the value of IPE.

Given the versatility inherent in IPE programmes used in the field of oncology, it is essential to develop a comprehensive evaluation strategy through the active involvement of professionals, patients, and their representatives.^3 9^ This strategy should include formative and summative assessments to effectively monitor the programmes’ impact and identify crucial areas for improvement.^10^ As Wong *et al*^9^ emphasised, incorporating patient feedback is crucial in this process, ensuring that the educational programmes align with the needs and expectations of all relevant stakeholders.

The findings of this scoping review will inform a range of stakeholders, including healthcare professionals, patients, and policymakers. By mapping existing patient-centred IPE programmes and identifying gaps in the current research landscape, this review aims to provide a comprehensive overview of the strategies applied to implement and evaluate these programmes and offer actionable insights for enhancing interdisciplinary collaboration and patient outcomes in cancer care and other healthcare domains. Furthermore, the review will contribute to the identification of best practices and support the development of robust patient-centred IPE models that could be adapted to other healthcare fields, ultimately enhancing the quality of patient care.

### Objective

Through the review described in this protocol, we seek to map and synthesise the existing academic and non-academic literature on patient-centred IPE in oncology. We summarise what is known on this topic and identify gaps to explore new pathways for improving interdisciplinary training in cancer care. By examining both qualitative and quantitative studies, we aim to delve into the implementation methods, impact, and extent of interdisciplinarity, as well as the evaluation strategies used within these programmes. This will enable us to better comprehend the importance of patient-centred and interdisciplinary approaches in cancer care to enhance the quality of treatment and overall patient outcomes.

This scoping review is part of the larger INTERACT-EUROPE 100 project, an initiative aimed at enhancing interdisciplinary collaboration in cancer care across Europe. Funded by the European Union under the EU4Health programme, INTERACT-EUROPE 100 will implement a comprehensive interspecialty training programme for healthcare professionals across various disciplines involved in cancer care. By fostering a deeper understanding of roles and competencies within interdisciplinary teams, this project seeks to improve the quality of cancer care by promoting a truly collaborative and patient-centred approach.

### Research question

How are patient-centred IPE programmes or curricula implemented in the field of cancer care, and what strategies are used to evaluate their impact and effectiveness?

## Methods and analysis

### Eligibility criteria

This scoping review will be conducted in accordance with the Joanna Briggs Institute (JBI) methodology for scoping reviews, which provides a comprehensive framework to ensure methodological rigour and reproducibility. The key steps include (1) identification of the research question, (2) identification of relevant studies, (3) study selection, (4) data charting, (5) collation, summarisation and reporting of results and (6) stakeholder consultation.^11^ These steps ensure that all relevant evidence is systematically identified and synthesised to address the scoping review objectives.

The following criteria were chosen to ensure that the planned review remains focused, relevant and comprehensive in evaluating the role and effectiveness of patient-centred IPE programmes in oncology:

- Inclusion criteria:
  - Original studies (qualitative or quantitative approach).
  - Professional education (eg, continuing education).
  - Interprofessionality/interdisciplinarity (ie, professionals from at least two occupations collaborating).
  - Patient-centredness.
- Exclusion criteria:

- Wrong publication type:
  - Non-original research (reviews, protocols, etc).
  - Letters, editorials, comments, etc.
  - Abstracts.
- Wrong topic:
  - Non-oncology.
  - Non-education.
  - Non-interdisciplinary.

### Types of sources

This scoping review will include a variety of study designs, such as experimental and quasi-experimental studies, including randomised controlled trials, non-randomised controlled trials and pre–post studies. We will also incorporate prospective and retrospective cohort studies, case–control studies and analytical cross-sectional studies. In addition, descriptive observational studies, such as case series, individual case reports and descriptive cross-sectional studies, will be eligible for inclusion.

To ensure comprehensive insights beyond quantitative data, we will include qualitative studies that provide detailed information on factors that facilitate or impede interdisciplinary collaboration and patient-centred care in oncology. Reviews that align with our eligibility criteria and offer relevant literature synthesis will also be considered. By including this diverse range of study designs, we aim to thoroughly explore the landscape of IPE and patient-centred practices in cancer care.

### Search strategy

After an initial limited search of MEDLINE (PubMed) and Scopus (Elsevier) to identify relevant articles, the titles and abstracts of these articles, along with the index terms used to describe them, will be used to develop a comprehensive search strategy for CENTRAL (Cochrane Library), CINAHL (EBSCO), Embase (Elsevier), MEDLINE (PubMed), PsycInfo (APA), Scopus (Elsevier) and Web of Science (Clarivate). The search strategy will be adapted for each database. The search will be performed on 11 September 2024 and coverd the above databases without restricting relevant studies’ language or date range. Sources of unpublished studies and grey literature, such as trial registries and grey literature databases (eg, OpenGrey, OpenDOAR), will also be searched. The complete search strategy can be found on Open Science Framework (OSF)Registries and in supplementary material 1.^12^

### Study and source of evidence selection

After the database search, all identified citations will be inserted into EndNote V.20 (Clarivate Analytics, Pennsylvania, USA), and duplicates will be removed automatically and manually. Then, two teams, each comprising two reviewers, will screen the titles and abstracts for relevant sources concerning the eligibility criteria and retrieve the full texts of selected studies. Five reviewers will assess the full-text eligibility, and discussion rounds will be held to resolve any conflicting decisions. The reasons for excluding sources and the final number of included results will be reported in the final scoping review and presented in a flow chart as outlined by the Preferred Reporting Items for Systematic Reviews and Meta-Analyses Extension for Scoping Reviews.^11^

### Data extraction

The data extraction tool will include information about source details, characteristics and results extraction following the guidance of the JBI methodology for scoping reviews. Specifically, the data extraction table will contain the following specific information:

1. Names of the author(s).
2. Publication year.
3. Country/region of origin or publication.
4. Objectives or purpose of the study/report.
5. Details on the patient population:
  - Demographic information.
  - Diagnosis/cancer type.
  - Sample size.
6. Details on the professional population:
  - Demographic information.
  - Occupation.
  - Sample size.
7. Methodology.
8. Setting of intervention.
9. Short description of intervention:
  - Type of intervention.
  - Duration of intervention.
10. End-points/dimensions/learning objectives.
11. Short description of intervention evaluation processes.
12. Key results/effects of intervention.

### Data analysis and presentation

By analysing the data collected through our data extraction framework, we will gain insights into the existing body of research on interdisciplinary collaboration and patient-centred care through IPE programmes in oncology. Moreover, the analysis will help identify knowledge gaps that, to date, have not received sufficient attention and may require further investigation. The findings will be presented in an appropriate visual and aggregate format, with a table summarising the descriptive results of the scoping review for each included source of evidence. Additionally, we will provide a narrative synthesis of the findings, discussing their relevance to our specific objectives and research questions.

## Patient and public involvement

Not applicable as this protocol is describing a planned scoping review that will not include participants.

## Ethics and dissemination

Ethical approval is not necessary for this study, as it will be a retrospective review of publicly available evidence sources and does not include collecting primary data. The findings of the scoping review will be disseminated through publications in peer-reviewed journals and presentations at symposia and conferences. To ensure that the review findings reach relevant stakeholders, a dissemination strategy will be developed later in the review process. The scoping review described in this protocol has been pre-registered in the OSF Registries.^12^

## supplementary material

10.1136/bmjopen-2024-089909 online supplemental file 1

## References

[1] World health organization (2022). Cancer. https://www.who.int/news-room/fact-sheets/detail/cancer.

[2] Borrill CS, Carletta J, Carter AJ (2013). The effectiveness of health care teams in the national health service. https://web.archive.org/web/20180721120857id_/http://homepages.inf.ed.ac.uk/jeanc/DOH-final-report.pdf.

[3] Aebersold ML, Kraft S, Farris KB (2021). Evaluation of an Interprofessional Training Program to Improve Cancer Drug Therapy Safety. *JCO Oncol Pract*.

[4] Interprofessional Education Collaborative (2016). Core competencies for interprofessional collaborative practice: 2016 update. https://ipec.memberclicks.net/assets/2016-Update.pdf.

[5] Reeves S, Fletcher S, Barr H (2016). A BEME systematic review of the effects of interprofessional education: BEME Guide No. 39. Med Teach.

[6] Zwarenstein M, Goldman J, Reeves S (2009). Interprofessional collaboration: effects of practice-based interventions on professional practice and healthcare outcomes. Cochrane Database Syst Rev.

[7] Barr H (2008). Effective interprofessional education: argument, assumption and evidence (Promoting Partnership for Health).

[8] Papadakos CT, Stringer T, Papadakos J (2021). Effectiveness of a Multiprofessional, Online and Simulation-Based Difficult Conversations Training Program on Self-Perceived Competence of Oncology Healthcare Provider Trainees. J Cancer Educ.

[9] Wong E, Mavondo F, Fisher J (2020). Patient feedback to improve quality of patient-centred care in public hospitals: a systematic review of the evidence. BMC Health Serv Res.

[10] Steinert Y (2005). Learning together to teach together: interprofessional education and faculty development. J Interprof Care.

[11] Tricco AC, Lillie E, Zarin W (2018). PRISMA Extension for Scoping Reviews (PRISMA-ScR): Checklist and Explanation. Ann Intern Med.

[12] Fragner T, Hama H, Šerifović A (2024). Patient-centered interprofessional education in cancer care: a systematic scoping review. BMC Med Educ.

