# Supplementary material for: Patient-centred interprofessional education in cancer care: a scoping review protocol
Source: BMJ Open. 2025 Feb 18;15(2):e089909. doi: 10.1136/bmjopen-2024-089909 (PMC11836791; doi:10.1136/bmjopen-2024-089909)
Supplement: online supplemental file 1 [file bmjopen-15-2-s001.docx]

**Search strategy for CENTRAL (Cochrane Library)**

|  | **#** | **Query** |
| --- | --- | --- |
| **patient centered** | 1 | patient-centered care/ or patient navigation/ |
|  | 2 | ("patient centred*" or "patient centered*" or "patient focus*" or "person centred*" or "person centered*" or "person focus*" or "client centred*" or "client centered*" or "client focus*").ti,ab,kw. |
|  | 3 | ("personalized medicine" or "personalised medicine" or "individualized medicine" or "individualised medicine").ti,ab,kw. |
|  | 4 | 1 OR 2 OR 3 |
| **multi-disciplinary** | 5 | exp Interprofessional Relations/ |
|  | 6 | (multiprofession* or "multi profession*" or multidisciplinar* or "multi disciplinar*" or interdisciplin* or "inter disciplin*" or interprofession* or "inter profession*" or collaborat* or cooperat* or "co operat*" or crossdisciplinar* or "cross disciplinar*" or multispecial* or "multi special*" or "team based" or "cross functional" or crossfunctional or transdisciplin* or "trans disciplin*" or interspecial* or "inter special*" or transsector* or "trans sector*").ti,ab,kw. |
|  | 7 | 5 OR 6 |
|  | 8 | 4 AND 7 |
| **cancer** | 9 | exp Neoplasms/ |
|  | 10 | exp Medical Oncology/ |
|  | 11 | (neoplas* or cancer* or oncolog* or tumor* or tumour* or carcino* or malignan*).ti,ab,kw. |
|  | 12 | 9 OR 10 OR 11 |
|  | 13 | 8 AND 12 |
| **education** | 14 | exp Education/ NOT exp Education, Nonprofessional/ |
|  | 15 | (educat* or curricul* or train* or learn* or teach* or course* or seminar* or workshop* or program* or IPE* or skill* or framework*).ti,ab,kw. |
|  | 16 | 14 OR 15 |
|  | 17 | 13 AND 16 |
| **NOT AI** | 18 | exp artificial intelligence/ |
|  | 19 | ("artificial intelligen*" or AI or "machine learning*").ti,ab,kw. |
|  | 20 | 18 OR 19 |
|  | **21** | **17 NOT 20** |

**Search strategy for CINAHL (Ebsco)**

|  | **#** | **Query** |
| --- | --- | --- |
|  | S24 | S20 NOT S23 |
| **NOT AI** | S23 | S21 OR S22 |
|  | S22 | TI ( "artificial intelligen*" or AI or "machine learning*" ) OR AB ( "artificial intelligen*" or AI or "machine learning*" ) |
|  | S21 | (MH "Artificial Intelligence+") |
|  | S20 | S14 AND S19 |
| **education** | S19 | S17 OR S18 |
|  | S18 | TI ( educat* or curricul* or train* or learn* or teach* or course* or seminar* or workshop* or program* or IPE* or skill* or framework* ) OR AB ( educat* or curricul* or train* or learn* or teach* or course* or seminar* or workshop* or program* or IPE* or skill* or framework* ) |
|  | S17 | S15 NOT S16 |
|  | S16 | (MH "Education, Nonprofessional+") |
|  | S15 | (MH "Education+") |
|  | S14 | S9 AND S13 |
| **cancer** | S13 | S10 OR S11 OR S12 |
|  | S12 | TI ( neoplas* or cancer* or oncolog* or tumor* or tumour* or carcino* or malignan* ) OR AB ( neoplas* or cancer* or oncolog* or tumor* or tumour* or carcino* or malignan* ) |
|  | S11 | (MH "Oncology+") |
|  | S10 | (MH "Neoplasms+") |
|  | S9 | S5 AND S8 |
| **multi-professional** | S8 | S6 OR S7 |
|  | S7 | TI ( multiprofession* or "multi profession*" or multidisciplinar* or "multi disciplinar*" or interdisciplin* or "inter disciplin*" or interprofession* or "inter profession*" or collaborat* or cooperat* or "co operat*" or crossdisciplinar* or "cross disciplinar*" or multispecial* or "multi special*" or "team based" or "cross functional" or crossfunctional or transdisciplin* or "trans disciplin*" or interspecial* or "inter special*" or transsector* or "trans sector*" ) OR AB ( multiprofession* or "multi profession*" or multidisciplinar* or "multi disciplinar*" or interdisciplin* or "inter disciplin*" or interprofession* or "inter profession*" or collaborat* or cooperat* or "co operat*" or crossdisciplinar* or "cross disciplinar*" or multispecial* or "multi special*" or "team based" or "cross functional" or crossfunctional or transdisciplin* or "trans disciplin*" or interspecial* or "inter special*" or transsector* or "trans sector*" ) |
|  | S6 | (MH "Interprofessional Relations+") |
| **patient centered** | S5 | S1 OR S2 OR S3 OR S4 |
|  | S4 | TI ( "personalized medicine" OR "personalised medicine" OR "individualized medicine" OR "individualised medicine" ) OR AB ( "personalized medicine" OR "personalised medicine" OR "individualized medicine" OR "individualised medicine" ) |
|  | S3 | TI ( "patient centred*" or "patient centered*" or "patient focus*" or "person centred*" or "person centered*" or "person focus*" or "client centred*" or "client centered*" or "client focus*" ) OR AB ( "patient centred*" or "patient centered*" or "patient focus*" or "person centred*" or "person centered*" or "person focus*" or "client centred*" or "client centered*" or "client focus*" ) |
|  | S2 | (MH "Patient Navigation") |
|  | S1 | (MH "Patient Centered Care") |

**Search strategy for Embase (Elsevier)**

|  | **#** | **Query** |
| --- | --- | --- |
| **patient centered** | 1 | patient centred*':ti,ab,kw OR 'patient centered*':ti,ab,kw OR 'patient focus*':ti,ab,kw OR 'person centred*':ti,ab,kw OR 'person centered*':ti,ab,kw OR 'person focus*':ti,ab,kw OR 'client centred*':ti,ab,kw OR 'client centered*':ti,ab,kw OR 'client focus*':ti,ab,kw |
|  | 2 | personalized medicine':ti,ab,kw OR 'personalised medicine':ti,ab,kw OR 'individualized medicine':ti,ab,kw OR 'individualised medicine':ti,ab,kw |
|  | 3 | 1 OR 2 |
| **multi-disciplinary** | 4 | interdisciplinary communication'/exp |
|  | 5 | multidisciplinary team'/exp |
|  | 6 | multiprofession*:ti,ab,kw OR 'multi profession*':ti,ab,kw OR multidisciplinar*:ti,ab,kw OR 'multi disciplinar*':ti,ab,kw OR interdisciplin*:ti,ab,kw OR 'inter disciplin*':ti,ab,kw OR interprofession*:ti,ab,kw OR 'inter profession*':ti,ab,kw OR collaborat*:ti,ab,kw OR cooperat*:ti,ab,kw OR 'co operat*':ti,ab,kw OR crossdisciplinar*:ti,ab,kw OR 'cross disciplinar*':ti,ab,kw OR multispecial*:ti,ab,kw OR 'multi special*':ti,ab,kw OR 'team based':ti,ab,kw OR 'cross functional':ti,ab,kw OR crossfunctional:ti,ab,kw OR transdisciplin*:ti,ab,kw OR 'trans disciplin*':ti,ab,kw OR interspecial*:ti,ab,kw OR 'inter special*':ti,ab,kw OR transsector*:ti,ab,kw OR 'trans sector*':ti,ab,kw |
|  | 7 | 4 OR 5 OR 6 |
|  | 8 | 3 AND 7 |
| **cancer** | 9 | malignant neoplasm'/exp |
|  | 10 | oncology'/exp |
|  | 11 | neoplas*:ti,ab,kw OR cancer*:ti,ab,kw OR oncolog*:ti,ab,kw OR tumor*:ti,ab,kw OR tumour*:ti,ab,kw OR carcino*:ti,ab,kw OR malignan*:ti,ab,kw |
|  | 12 | 9 OR 10 OR 11 |
|  | 13 | 8 AND 12 |
| **education** | 14 | 'education'/exp NOT 'health education'/exp |
|  | 15 | educat*:ti,ab,kw OR curricul*:ti,ab,kw OR train*:ti,ab,kw OR learn*:ti,ab,kw OR teach*:ti,ab,kw OR course*:ti,ab,kw OR seminar*:ti,ab,kw OR workshop*:ti,ab,kw OR program*:ti,ab,kw OR ipe*:ti,ab,kw OR skill*:ti,ab,kw OR framework*:ti,ab,kw |
|  | 16 | 14 OR 15 |
|  | 17 | 13 AND 16 |
| **NOT AI** | 18 | artificial intelligence'/exp |
|  | 19 | machine learning'/exp |
|  | 20 | 'artificial intelligen*':ti,ab,kw OR ai:ti,ab,kw OR 'machine learning*':ti,ab,kw |
|  | 21 | 18 OR 19 OR 20 |
|  | **22** | **17 NOT 21** |

**Search strategy for MEDLINE (PubMed)**

|  | **#** | **Query** |
| --- | --- | --- |
| **patient centered** | 1 | patient-centered care/ or patient navigation/ |
|  | 2 | ("patient centred*" or "patient centered*" or "patient focus*" or "person centred*" or "person centered*" or "person focus*" or "client centred*" or "client centered*" or "client focus*").ti,ab,kf. |
|  | 3 | ("personalized medicine" or "personalised medicine" or "individualized medicine" or "individualised medicine").ti,ab,kf. |
|  | 4 | 1 OR 2 OR 3 |
| **multi-disciplinary** | 5 | exp Interprofessional Relations/ |
|  | 6 | (multiprofession* or "multi profession*" or multidisciplinar* or "multi disciplinar*" or interdisciplin* or "inter disciplin*" or interprofession* or "inter profession*" or collaborat* or cooperat* or "co operat*" or crossdisciplinar* or "cross disciplinar*" or multispecial* or "multi special*" or "team based" or "cross functional" or crossfunctional or transdisciplin* or "trans disciplin*" or interspecial* or "inter special*" or transsector* or "trans sector*").ti,ab,kf. |
|  | 7 | 5 OR 6 |
|  | 8 | 4 AND 7 |
| **cancer** | 9 | exp Neoplasms/ |
|  | 10 | exp Medical Oncology/ |
|  | 11 | (neoplas* or cancer* or oncolog* or tumor* or tumour* or carcino* or malignan*).ti,ab,kf. |
|  | 12 | 9 OR 10 OR 11 |
|  | 13 | 8 AND 12 |
| **education** | 14 | exp Education/ NOT exp Education, Nonprofessional/ |
|  | 15 | (educat* or curricul* or train* or learn* or teach* or course* or seminar* or workshop* or program* or IPE* or skill* or framework*).ti,ab,kf. |
|  | 16 | 14 OR 15 |
|  | 17 | 13 AND 16 |
| **NOT AI** | 18 | exp artificial intelligence/ |
|  | 19 | ("artificial intelligen*" or AI or "machine learning*").ti,ab,kf. |
|  | 20 | 18 OR 19 |
|  | **21** | **17 NOT 20** |

**Seach strategy for PsycInfo (APA)**

|  | **#** | **Query** |
| --- | --- | --- |
| **patient centered** | 1 | patient-centered care.mh. |
|  | 2 | patient navigation.mh. |
|  | 3 | ("patient centred*" or "patient centered*" or "patient focus*" or "person centred*" or "person centered*" or "person focus*" or "client centred*" or "client centered*" or "client focus*").ti,ab,hw,id. |
|  | 4 | ("personalized medicine" or "personalised medicine" or "individualized medicine" or "individualised medicine").ti,ab,hw,id. |
|  | 5 | 1 or 2 or 3 or 4 |
| **multi-professional** | 6 | Interprofessional Relations.mh. |
|  | 7 | (multiprofession* or "multi profession*" or multidisciplinar* or "multi disciplinar*" or interdisciplin* or "inter disciplin*" or interprofession* or "inter profession*" or collaborat* or cooperat* or "co operat*" or crossdisciplinar* or "cross disciplinar*" or multispecial* or "multi special*" or "team based" or "cross functional" or crossfunctional or transdisciplin* or "trans disciplin*" or interspecial* or "inter special*" or transsector* or "trans sector*").ti,ab,hw,id. |
|  | 8 | 6 or 7 |
|  | 9 | 5 and 8 |
| **cancer** | 10 | neoplasms.mh. |
|  | 11 | medical oncology.mh. |
|  | 12 | (neoplas* or cancer* or oncolog* or tumor* or tumour* or carcino* or malignan*).ti,ab,hw,id. |
|  | 13 | 10 or 11 or 12 |
|  | 14 | 9 and 13 |
| **education** | 15 | education.mh. |
|  | 16 | education, nonprofessional.mh. |
|  | 17 | 15 not 16 |
|  | 18 | (educat* or curricul* or train* or learn* or teach* or course* or seminar* or workshop* or program* or IPE* or skill* or framework*).ti,ab,hw,id. |
|  | 19 | 17 or 18 |
|  | 20 | 14 and 19 |
| **NOT AI** | 21 | artificial intelligence.mh. |
|  | 22 | ("artificial intelligen*" or AI or "machine learning*").ti,ab,hw,id. |
|  | 23 | 21 or 22 |
|  | **24** | **20 not 23** |

**Search strategy for Scopus (Elsevier)**

|  | **#** | **Query** |
| --- | --- | --- |
| **patient centered** | 1 | TITLE-ABS-KEY ( "patient centred*" OR "patient centered*" OR "patient focus*" OR "person centred*" OR "person centered*" OR "person focus*" OR "client centred*" OR "client centered*" OR "client focus*" ) |
|  | 2 | TITLE-ABS-KEY ( "personalized medicine" OR "personalised medicine" OR "individualized medicine" OR "individualised medicine" ) |
|  | 3 | 1 OR 2 |
| **multi-professional** | 4 | TITLE-ABS-KEY ( multiprofession* OR "multi profession*" OR multidisciplinar* OR "multi disciplinar*" OR interdisciplin* OR "inter disciplin*" OR interprofession* OR "inter profession*" OR collaborat* OR cooperat* OR "co operat*" OR crossdisciplinar* OR "cross disciplinar*" OR multispecial* OR "multi special*" OR "team based" OR "cross functional" OR crossfunctional OR transdisciplin* OR "trans disciplin*" OR interspecial* OR "inter special*" OR transsector* OR "trans sector*" ) |
|  | 5 | 3 AND 4 |
| **cancer** | 6 | TITLE-ABS-KEY ( neoplas* OR cancer* OR oncolog* OR tumor* OR tumour* OR carcino* OR malignan* ) |
|  | 7 | 5 AND 6 |
| **education** | 8 | TITLE-ABS-KEY ( educat* OR curricul* OR train* OR learn* OR teach* OR course* OR seminar* OR workshop* OR program* OR ipe* OR skill* OR framework* ) |
|  | 9 | 7 AND 8 |
| **NOT AI** | 10 | TITLE-ABS-KEY ( "artificial intelligen*" OR ai OR "machine learning*" ) |
|  | **11** | **9 NOT 10** |

**Search strategy for Web of Science (Clarivate)**

|  | **#** | **Query** |
| --- | --- | --- |
| **patient centered** | 1 | TS=("patient centred*" or "patient centered*" or "patient focus*" or "person centred*" or "person centered*" or "person focus*" or "client centred*" or "client centered*" or "client focus*") |
|  | 2 | TS=("personalized medicine" or "personalised medicine" or "individualized medicine" or "individualised medicine") |
|  | 3 | 1 OR 2 |
| **multi-disciplinary** | 4 | TS=(multiprofession* or "multi profession*" or multidisciplinar* or "multi disciplinar*" or interdisciplin* or "inter disciplin*" or interprofession* or "inter profession*" or collaborat* or cooperat* or "co operat*" or crossdisciplinar* or "cross disciplinar*" or multispecial* or "multi special*" or "team based" or "cross functional" or crossfunctional or transdisciplin* or "trans disciplin*" or interspecial* or "inter special*" or transsector* or "trans sector*") |
|  | 5 | 3 AND 4 |
| **cancer** | 6 | TS=(neoplas* or cancer* or oncolog* or tumor* or tumour* or carcino* or malignan*) |
|  | 7 | 5 AND 6 |
| **education** | 8 | TS=(educat* or curricul* or train* or learn* or teach* or course* or seminar* or workshop* or program* or IPE* or skill* or framework*) |
|  | 9 | 7 AND 8 |
| **NOT AI** | 10 | TS=("artificial intelligen*" or AI or "machine learning*") |
|  | **11** | **9 NOT 10** |
