# Supplementary material for: Patient-centred interprofessional education in cancer care: a scoping review protocol
Source: BMJ Open. 2025 Feb 18;15(2):e089909. doi: 10.1136/bmjopen-2024-089909 (PMC11836791; doi:10.1136/bmjopen-2024-089909)
Supplement: Supplementary file 2 [file bmjopen-2024-089909.reviewer_comments.pdf]

PEER REVIEW HISTORY

BMJ Open publishes all reviews undertaken for accepted manuscripts. Reviewers are asked to complete a checklist review form and are provided with free text boxes to elaborate on their assessment. These free text comments are reproduced below.

ARTICLE DETAILS

Title (Provisional)

Patient-centred Interprofessional Education in Oncology: A Scoping Review Protocol

Authors

Šerifović, Adis; Fragner, Tobias; Hama, Honja; Kirchheiner, Kathrin; Grabovac, Igor

---

VERSION 1 - REVIEW

**Reviewer**	**1**
**Name**	**Lemonde, Manon**
**Affiliation**	**Ontario Tech University, Faculty of Health Sciences**
**Date**	**10-Jul-2024**
**COI**	**n/a**

This is a very well written manuscript

and only requires minor revisions.

The authors

**** **The reviewer provided a marked copy with additional comments. Please contact the publisher for full details.** ****

**Reviewer**	**2**
**Name**	**Consolo, Letteria**
**Affiliation**	**University of Rome Tor Vergata**
**Date**	**09-Sep-2024**
**COI**	**no conflicts of interest**

Overall Assessment:

This scoping protocol presents an important and compelling objective, but there are key areas where clarification and refinement would significantly improve its effectiveness and impact.

STRENGTHS AND LIMITATIONS OF THE STUDY

In the strengths section, it is crucial to emphasize the broader implications of the study's findings for stakeholders, such as healthcare professionals, patients, and institutions.

INTRODUCTION

To enhance the introduction, provide a more detailed explanation of IPE, specifically outlining how this educational approach supports better patient care not only within oncology but also in other medical specialties. Furthermore, includes a synthesis of existing literature on IPE programs in non-oncological settings. Discuss whether comparable training initiatives are found in other fields, specifying their duration, content, and structure, and how these insights could inform and improve oncology-related IPE programs.

METHODS (Page 3 of 15)

In the methods section, include a paragraph explicitly stating that the scoping review will be conducted following the Joanna Briggs Institute (JBI) guidelines. Clearly outline the steps of the review, which should be elaborated upon in subsequent subsections for example::

Identification of the Research Question

Identification of Relevant Studies

Study Selection

Data Charting

Collation, Summarization, and Reporting of Results

Stakeholder consultation

It is essential to explain how each of these steps aligns with the JBI's robust methodological framework, ensuring the review is conducted with transparency, reliability, and reproducibility.

ADDITIONAL NOTE (Page 3 of 15, Line 53)

The sentence, "This effort is part of the INTERACT-EUROPE 100 project, which focuses on implementing an inter-speciality training programme for healthcare professionals across different disciplines involved in cancer care," should also be highlighted in the strengths section. This underscores the relevance of the project, as it addresses critical elements of interdisciplinary healthcare education and has the potential for far-reaching applications beyond the scope of oncology.

DISCUSSION

Include a discussion section, in which the expected results of the scoping review can be discussed in more detail. Highlight how your research will contribute to identifying and mapping existing IPE programs in the oncology field. This mapping is expected to provide a comprehensive overview of current IPE practices and address gaps in healthcare education. Additionally, discuss how these findings could influence the development and integration of more robust, interdisciplinary training programs, both within oncology and potentially across other healthcare domains.

VERSION 1 - AUTHOR RESPONSE

Reviewer 1 comments	Authors' responses	Page(s) / Paragraph(s)
This is a very well written manuscript and only requires minor revisions.	Thank you for your positive feedback. We are pleased that you found our manuscript well-written. We have addressed all the comments and made amendments as suggested.	

Reviewer 2 comments	Authors' responses	Page(s) / Paragraph(s)
Overall Assessment: This scoping protocol presents an important and compelling objective but there are key areas where clarification and refinement would significantly improve its effectiveness and impact.	Thank you for your overall assessment and constructive feedback. We have carefully reviewed your comments and have made adjustments to clarify and refine the relevant sections as recommended.	
In the strengths section it is crucial to emphasize the broader implications of the study's findings for stakeholders such as healthcare professionals, patients, and institutions.	Thank you for this suggestion. However, in accordance with the editor's request, the strengths section has been revised to focus exclusively on methodological aspects. We have therefore included the broader implications in a newly added discussion section based on your last comment, where it is more appropriate to address the impact of the study on various stakeholders.<br>The added section reads as follows:	Page 5, Section: Discussion

	DISCUSSION<br>*The findings of this scoping review will inform a range of stakeholders, including healthcare professionals, patients, and policymakers. By mapping existing patient-centred IPE programs and identifying gaps in the current research landscape, this review aims to provide a comprehensive overview of the strategies applied to implement and evaluate these programmes and offer actionable insights for enhancing interdisciplinary collaboration and patient outcomes in cancer care and other healthcare domains. Furthermore, the review will contribute to the identification of best practices and support the development of robust patient-centred IPE models that could be adapted to other healthcare fields, ultimately enhancing the quality of patient care.*	
To enhance the introduction, provide a more detailed explanation of IPE, specifically outlining how this educational approach supports better patient care not only within oncology but also in other medical specialties. Furthermore, include a synthesis of existing literature on IPE programs in non-oncological settings. Discuss whether comparable training initiatives are found in other fields, specifying their duration, content, and structure, and how these insights could inform and improve oncology-related IPE	Thank you for this detailed comment. We have revised the introduction to include a more comprehensive explanation of IPE and its benefits across different medical specialties. We have also incorporated a synthesis of relevant literature on IPE programmes in non-oncological contexts, discussing their duration, content, and structure. This expanded introduction section now outlines how these insights can be adapted to improve IPE programs	Page 2, Section: Introduction

programs.	in cancer care.<br><br>Our amendments in the introduction section read as follows:<br><br>*IPE has been identified as a key strategy for improving collaboration among healthcare workers. It enhances collaborative learning and teamwork across various healthcare disciplines, improving patient outcomes in fields such as primary care, paediatrics, and surgery [5]. Non-oncological IPE programs, such as those implemented in surgery and paediatrics, have demonstrated reduced medical errors, improved communication, and better patient safety [6]. These programs typically vary in structure and duration, ranging from short-term workshops to long-term integrated training, depending on the speciality and the specific learning objectives. Incorporating these insights into oncology-related IPE programs can help improve the interdisciplinary collaboration needed for complex cancer care.*	
In the methods section, include a paragraph explicitly stating that the scoping review will be conducted following the Joanna Briggs Institute (JBI) guidelines. Clearly outline the steps of the review, which should be elaborated upon in subsequent subsections, for example: Identification of the Research	Thank you for this comment. We have added a new paragraph in the methods section that explicitly describes each step of the scoping review in accordance with the JBI guidelines. The steps include: Identification of the Research Question, Identification of Relevant Studies, Study Selection,	Page 3, Section: Methods and Analysis

Question, Identification of Relevant Studies, Study Selection, Data Charting, Collation, Summarization, and Reporting of Results, Stakeholder Consultation. It is essential to explain how each of these steps aligns with the JBI's robust methodological framework, ensuring the review is conducted with transparency, reliability, and reproducibility.	Data Charting, Collation, Summarization and Reporting of Results, and Stakeholder Consultation. Each step is aligned with the JBI's framework to ensure transparency, reliability, and reproducibility. We have changed the main text as follows:<br><br>*This scoping review will be conducted in accordance with the Joanna Briggs Institute (JBI) methodology for scoping reviews, which provides a comprehensive framework to ensure methodological rigour and reproducibility. The key steps include (1) Identification of the Research Question, (2) Identification of Relevant Studies, (3) Study Selection, (4) Data Charting, (5) Collation, Summarization, and Reporting of Results, and (6) Stakeholder Consultation [11]. These steps ensure that all relevant evidence is systematically identified and synthesised to address the scoping review objectives.*	
The sentence 'This effort is part of the INTERACT-EUROPE 100 project which focuses on implementing an inter-speciality training programme for healthcare professionals across different disciplines involved in cancer care' should also be highlighted in the strengths section. This underscores the relevance of the project as it addresses critical	Thank you for your comment and for recognizing the potential of the INTERACT-EUROPE 100 project. While we appreciate your suggestion to highlight this in the strengths section, we are hesitant to do so due to guidance from the editor. In their previous feedback, the editor specifically advised against including general	Page 3, Section: Introduction

elements of interdisciplinary healthcare education and has the potential for far-reaching applications beyond the scope of oncology.	information about our planned scoping review in the strengths section. We are aiming to adhere closely to the editor's recommendations to ensure the manuscript aligns with their expectations.<br><br>However, we have ensured that the sentence you mentioned remains prominent in the manuscript's introduction to clearly convey the project's context and relevance:<br><br>*This scoping review is part of the larger INTERACT-EUROPE 100 project, an initiative aimed at enhancing interdisciplinary collaboration in cancer care across Europe. Funded by the European Union under the EU4Health programme, INTERACT-EUROPE 100 will implement a comprehensive inter-specialty training program for healthcare professionals across various disciplines involved in cancer care. By fostering a deeper understanding of roles and competencies within multidisciplinary teams, this project seeks to improve the quality of cancer care by promoting a truly collaborative and patient-centred approach.*	
Include a discussion section in which the expected results of the scoping review can be discussed in more detail. Highlight how your research will contribute to identifying and	Thank you for this detailed suggestion. As depicted above, we have added a discussion section that outlines the expected contributions of our review, such as	Page 6, Section: Discussion

mapping existing IPE programs in the oncology field. This mapping is expected to provide a comprehensive overview of current IPE practices and address gaps in healthcare education. Additionally, discuss how these findings could influence the development and integration of more robust interdisciplinary training programs both within oncology and potentially across other healthcare domains.	mapping current patient-centred IPE programs, identifying gaps, and providing recommendations for future research. We also discuss how these findings could be used to inform the development of robust interdisciplinary training programmes within oncology and across other healthcare domains.<br>The added section reads as follows:<br><br>*DISCUSSION*<br>*The findings of this scoping review will inform a range of stakeholders, including healthcare professionals, patients, and policymakers. By mapping existing patient-centred IPE programs and identifying gaps in the current research landscape, this review aims to provide a comprehensive overview of the strategies applied to implement and evaluate these programmes and offer actionable insights for enhancing interdisciplinary collaboration and patient outcomes in cancer care and other healthcare domains. Furthermore, the review will contribute to the identification of best practices and support the development of robust patient-centred IPE models that could be adapted to other healthcare fields, ultimately enhancing the quality of patient care.*	

VERSION 2 - AUTHOR RESPONSE

Reviewer comments	Authors' responses	Page(s), Paragraph(s)
"Clarify what you mean by 'inception'."	Thank you for this comment. We have revised the manuscript and replaced the term 'inception' with the sentence:<br><br>*Each database will be searched without date restrictions.*	Page 1, Methods and Analysis
"What about impact to be congruent with previous wording"	We appreciate your attention to consistency in terminology. The wording has been revised throughout the manuscript.	Page 1, Methods and Analysis, and throughout manuscript
"of what? you did not say this word but mentioned before that interdisciplinary approaches are crucial 2 # concepts so please clarify"	Thank you for this observation. We have clarified the sentence by adding *"such as rigid hierarchies, lack of standardized communication protocols, and undefined interdisciplinary roles*".	Page 2, Introduction
"now new issue but based on what?"	Thank you for this comment. We have revised the sentence to provide additional clarity and context for the term "multi-faceted." The revised text now elaborates on the integration of physical, psychological, and social care, emphasizing personalized treatment plans tailored to the unique needs of patients and their families. This ensures that the use of "multi-faceted" is supported by specific examples that align with the interdisciplinary approach described in the manuscript.<br><br>*This includes physical, psychological, and social care, as well as personalized treatment plans that address the unique needs of each patient and their families.*	Page 2, Introduction
"Issues such as...? Can you clarify this sentence and add strong evidence."	Thank you for pointing this out. We have revised the manuscript to include additional evidence supporting the challenges of interdisciplinary approaches. For example, we referenced Aebersold et al. (2021) to illustrate how the lack of standardized communication	Page 2, Introduction

	protocols can lead to errors in chemotherapy administration.	
"Evidence please to support this first sentence."	Thank you for this comment. We have revised the sentence to include supporting evidence for the versatility of IPE programs and the necessity of comprehensive evaluation strategies. For example, Aebersold et al. (2021) demonstrated that the effectiveness of IPE programs depends on tailored evaluation frameworks that address specific interdisciplinary challenges and outcomes. Additionally, Wong et al. (2020) highlighted the importance of stakeholder engagement in ensuring the relevance and impact of evaluation methods.	Page 2, Introduction
"? Interdisciplinary"	Thank you, we changed the wording from multidisciplinary to interdisciplinary.	Page 3, Objective
"what about impact?"	Thank you for this comment. We have revised the research question to explicitly address the concept of "impact."	Page 3, research question
